# Age-Related Digital Divide during the COVID-19 Pandemic in China

**DOI:** 10.3390/ijerph182111285

**Published:** 2021-10-27

**Authors:** Yu Song, Chenfei Qian, Susan Pickard

**Affiliations:** 1 XIPU Institution, Xi’an Jiaotong-Liverpool University, Suzhou 215123, China; 2 Department of China Studies, Xi’an Jiaotong-Liverpool University, Suzhou 215123, China; chenfei.qian19@student.xjtlu.edu.cn; 3 Department of Sociology, Social Policy and Criminology, University of Liverpool, Liverpool L69 7ZA, UK; susan.pickard@liverpool.ac.uk

**Keywords:** older people, COVID-19 pandemic, digital divide, digital code

## Abstract

China has adopted a variety of digital technologies to effectively combat the unprecedented COVID-19 pandemic. The massive utilisation of digital technologies, however, to a great extent, magnifies the age-related digital divide. This paper aims to examine the impacts of the age-related digital divide on older adults in the context of the COVID-19 pandemic. Cases of three age-related digital divide scenarios, including older people taking public transportation, seeking medical care, as well as conducting digital transactions, are collected from Chinese official news outlets. The results indicate that the COVID-19 pandemic accelerates the pace of digital technology utilisation but exacerbates the age-related digital divide. Such an age-related digital divide has largely excluded older adults from both the real society and the virtual society. Older adults’ personal attitudes and motivations, as well as education and income, governmental policies, and family and social supports, are all major contributors to the severe impacts of the age-related digital divide on old adults during the pandemic. More measures should be adopted to bridge the age-related digital divide and build a senior-friendly e-society.

## 1. Introduction

As the COVID-19 pandemic has become an unprecedented global health crisis, governments worldwide have implemented a series of actions to control the spread of the virus. The lockdowns and self-isolations consequently entail a surge in the use of digital technologies. Various beneficiaries have taken advantage of using digital technologies for an accurate tracking and tracing routine, remote working and studying, timely seeking and sharing of information, and effectively maintaining daily life. There is no doubt that the COVID-19 pandemic has accelerated the pace of digital technology utilisation. Nonetheless, not everyone benefits equally from using digital technologies. Older adults are less likely to use digital technologies than younger groups in both developed and developing countries due to their higher digital illiteracy [1,2,3,4,5,6], low level of income [7,8,9], limited access to digital devices and the Internet [8,10,11,12], as well as technology anxiety or reluctant adoption of new technologies [13,14,15].

At the time of writing this paper, the COVID-19 pandemic in China is receding and stabilizing. China has managed to wrest control of its pandemic and reopen its economy [16]. To combat the coronavirus, China has rolled out perhaps the most ambitious, agile, and aggressive, yet most effective disease containment effort in history [17]. A variety of digital technologies are being used in the battle against the pandemic. Temperature screens with infrared thermometers are placed in airports, at railway and bus stations, and at ferry terminals. A nationwide health e-code system categorizes individuals into three colour groups based on their health status and travel history, and then determines whether they need to be quarantined [18]. Drones equipped with echoing loudspeakers and street camera systems are utilised in some cities to rebuke residents who are not following the rules in public places [16,18]. Tracking of movement, identification and surveillance of confirmed cases, manufacturing and distribution of diagnostic kits, dispatching of medical teams and volunteers, as well as governance of local communities all take full advantages of using digital technologies. Using digital technologies in the time of the crisis enables the Chinese government to respond to and effectively control the pandemic in a timely fashion.

However, the massive utilization of digital technologies during the COVID-19 pandemic without considering the age-related digital divide has produced several issues. It was frequently reported that older adults could not take public transportation, access medical care, or conduct commercial transactions because they did not have the required digital codes [19,20,21,22,23,24,25,26,27]. In light of these circumstances, this research aims to use age-related digital divide cases collected from the Chinese official news outlets to address the following questions: what are the impacts of the age-related digital divide on older adults? Additionally, what are the causes of such impacts? The rest of this paper is organized as follows. Section 2 introduces the analytical framework. Section 3 presents collected cases of three different scenarios, followed by Section 4 discussions and conclusions, and Section 5 policy implications and future research.

## 2. Analytical Framework

### 2.1. Digital Divide

The term digital divide first appeared in a report released by the National Telecommunications and Information Administration (NTIA), U.S. Department of Commerce, in the mid-1990s [28]. The digital divide originally refers to the gap between individuals who have access to the information communication technologies (ICTs) and those who do not have access to ICTs [29,30]. Those who had physical access to ICTs were perceived as being on the preferred side of the divide [31]. With the development of technology, this binary classification of ‘digital divide’—with or without access to ICTs—has been criticized by many researchers [28,32,33,34,35,36]. The term digital divide has gradually shifted from the physical access to the information communication technologies (the first level of digital divide), to how users use ICTs and what users use them for [37]. As highlighted by a large section of the literature, since the beginning of the 21st century, the research on the digital divide has progressed to a more sophisticated level that emphasizes the digital divide in terms of skills, knowledge, support quality and training opportunities [38,39,40,41,42,43,44,45,46,47,48], creating what has been defined by Hargittai as the second level of the digital divide [48]. Although the internet connection rates have been increasing at a significantly high speed and the divides of physical access to ICTs are narrowing globally, scholars of the second-level digital divide have concluded that the divide in digital skills continue to expand even after physical access to ICTs is universal [49,50,51,52,53,54,55]. Age, income, education level and job experience are indicated as the most influential factors for various users acquiring digital skills and knowledge [6,7,56,57,58,59]. More importantly, in the post-typographic society, digital information has pervaded all aspects of our life. Various information is produced, distributed and received through electronic media. ICTs, along with other digital tools, become the basic utilities for individuals to receive information. The importance of media and information literacy is increasing. With the advent of a new multimedia environment as a result of media convergence and multiplication of media platforms, the ability to access, understand, and critically evaluate media, and further carefully retrieve and select information from the media plays a vital role in better surviving in a digital society [60,61]. Media and information illiteracy is a major determinant of the digital divide [44,62,63,64].

More recently, the digital divide discourse has paid more attention to the different outcomes and consequences of digital technology utilisation, which is labelled the third level of digital divide [31,49,65]. An increasing number of studies in the field of digital divide have been conducted to investigate different consequences and unequal returning benefits deriving from accessing and using various digital technologies [37,65]. The third level of digital divide is strongly tied with different types of capital, especially social capital and cultural capital, and social, economic and cultural variables play significant roles in influencing the third level of digital divide [37,65,66,67,68,69,70]. In an information society, information is not only a primary product essential for individuals to survive, but also a positional product [71]. Obtaining, processing and using information reflects the socio-economic position and cultural capital of an individual and further determines his or her potential power in society [30]. For the information-poor population, lacking access to information as a result of the digital divide would entail social exclusion [72,73,74,75,76].

To sum up, the term digital divide is a multidimensional and dynamic concept which includes a set of complex divides [77]. Digital divides can be categorized into three levels in terms of access and coverage, utility and skills, and outcomes [78]. The first level of digital divide is the divide in physical access to the Internet and related information communication technologies. The second level of digital divide is the divide in digital skills and knowledge that required to effectively use ICTs, and the third level of digital divide is the different outcomes of using various digital technologies [30,31,37,48,49,78,79,80].

### 2.2. Age-Related Digital Divide

Older adults, one of the groups most represented in the information-poor population, are facing various difficulties in the process of rapid digitalization [81,82]. One issue worthy of note is their low adoption rate of digital devices and limited utilisation of digital applications [83,84,85]. Although older adults’ attitudes and motivations towards using information technology are patterned along the lines of age, gender, income, job experience, level of education, technology anxiety, marital status, health status, geographical location, and ethnicity [6,7,56,57,58,59,86,87,88,89,90,91,92,93,94], they are more likely to hold negative attitudes and low motivation towards the adoption of new technology [89,90,91,92]. Moreover, support from family, friends, and the community are essential for older adults to develop digital literacy and accept digital technologies, especially in unexpected situation [54,95,96,97,98].

Apart from the above-mentioned causes of the age-related divide, ICT device developers’ lack of consideration of older adults’ decline in cognitive functions will also lead to the widening of the digital divide [99,100,101,102]. As people age, their visual and aural acuity and accommodation will deteriorate and their response speed and cognition function will decline [103]. The lack of older-adult user-friendly ICT devices will exacerbate the age-related digital divide [83,84,85,86].

### 2.3. Age-Related Digital Divide during the COVID-19 Pandemic

The unprecedented COVID-19 pandemic has magnified the age-related digital divide. As the most vulnerable group, older adults bear both direct and indirect risks in the pandemic [1]. Recent empirical studies conducted in the Netherlands, America, and Germany have all found that older adults use the Internet less, resulting in a lack of critical information or necessary support, which has in turn further endangered their health situations and led to higher incidences of infection and mortality [94,104,105,106].

Chinese older adults also face challenges during the pandemic due to the age-related digital divide. To effectively control the spread of the virus, personal movement is strictly monitored and regulated by the government during the pandemic. To efficiently regulate personal movement, a digital health code was invented within a week in Hangzhou and soon applied across the nation. This is a smartphone-based digital code that classifies individuals’ health status into three colours. People are allowed to move in public only when they have been granted the valid green health code (see Table 1). During the pandemic, strict policies have been implemented nationwide to control the spread of the virus (see Table 2). For individuals who are from medium- or high-risk areas, nucleic tests and quarantine are required if he or she wants to travel. For those from low-risk areas, a valid green health code alone can be used as the travel certificate. In some areas, a valid pass code is also used as a travel permit, which is another digital code based on smartphones and is especially used in local areas. Valid codes are required when an individual enters any public area such as airports, bus stations, subway stations, supermarkets, restaurants, sports centres, museums, and parks.

In addition, many daily activities and routine events usually organized on site prior to the pandemic had to be organized online due to social distancing and public area closures. Most medical care institutions closed onsite appointment desks. Only online appointments were accepted in the hospitals. Patients could make an appointment either through the Internet or by telephone. Few medical care institutions offered onsite appointments, but a limited number of onsite appointments could only be obtained from self-service machines in hospitals. Again, a valid health code or pass code is needed to enter medical care institutions.

As filial piety plays a significant role in Chinese society, older adults have a long tradition of relying on their family members, particularly their children, for elder care. Along with this tradition, China’s various laws oblige children to support their parents both emotionally and finically [107]. Therefore, family members, in particular children, are older adults’ primary helpers during the pandemic.

## 3. Case Study Methodology

With approximately 988 million Internet users in 2020, China has the largest number of Internet users, as well as mobile Internet users (99.3 per cent of the total Internet users) [108]. With a good Internet infrastructure nationwide, various digital technologies are being applied across the country to prevent and control the COVID-19 pandemic. Among them, smartphone-based digital codes, including a health code (*jiankang ma)*, a payment code *(zhifu ma)* and a pass code (*tongxing ma)* are most widely adopted as licenses for individuals to take public transportation, book medical care services, conduct online shopping and other activities to maintain daily life.

However, there is a paradoxical situation that the population most affected by the pandemic is also the population least helped by the digital technology aiming to mitigate the negative effects. Among 988 million internet users, 109 million people were older adults aged 60 years and above [108]. Considering the total number of older adults aged 60 and above had reached 264 million at the end of 2020 [109], there were still 58.7 per cent of older adults who did not use the Internet for various reasons. During the time of the pandemic, older adults without access to digital devices and/or the Internet could not obtain various digital codes which were used as licenses for all sorts of activities. Without these licenses, they are trapped in this digital society, as digital codes are being utilised everywhere.

### 3.1. Three Most Affected Scenarios by Age-Related Digital Divide during the Pandemic

In order to effectively support older adults to address their difficulties in using the information technology during the pandemic and further adapt to the digital society, on 24 November 2020, General Office of the State Council of China issued a national policy ‘Implementation Plan for Practically Solving Difficulties of the Older People in Using Intelligent Technology’ [110]. In this plan, the Chinese government identified three scenarios as the top three scenarios in which older adults are most affected, namely transportation, medical care, and consumption.

### 3.2. Selection of Cases

During the COVID-19 pandemic, digital technologies have been massively utilized to control the spread of the virus. Although such extensive applications of digital technologies have been proved to be efficient and effective to serve the desired purposes, it was frequently reported by Chinese official news outlets that older adults experience various difficulties and challenges in their daily life, particularly due to the digital divide. It is important to investigate older adults’ difficult situations, as well as the causes and effects of the age-related digital divide during the pandemic.

However, due to the prevention and control regulations during the pandemic, it is extremely difficult for researchers to conduct fieldwork and interview older adults. We used the WiseResearch database for case collection. This database has collected over 3 billion archived news items, including printed media, online news, and social media in the Greater China region since 1998, with a daily increase of 3.5 million news items on average. We entered the keywords ‘older adult’, ‘smartphone’, ‘smart device’, ‘health code’, ‘pass code’, and ‘payment code’ in the top three Chinese official media in the WiseSearch database, namely, People.cn (Renmin Wang), Xinhuanet (Xinhua Wang) and GMW (Guangming Wang), which are official websites of People’s Daily, Xinhua News Agency, and Guangming Daily, respectively, to search news released between February 2020 and February 2021. We obtained 293 news reports covering age-related digital divide cases. We read all the reports and identified 9 cases by the following criteria: firstly, the case should be a full story and relevant to one of three scenarios under examination; secondly, the case should contain adequate scenario information for analysis (see Table 3).

## 4. Discussions and Conclusions

The age-related digital divide exists alongside the inevitable population ageing and rapid digitalization process [111,112]. However, the pandemic has exacerbated such an aged-related digital divide. By drawing on nine representative cases of the three most affected scenarios covered by the leading official media in China, the research examines the impacts of the age-related digital divide on older adults during the COVID-19 pandemic and analyses the causes of such impacts.

### 4.1. The Impacts of Age-Related Digital Divide on the Older Adults during the Pandemic

When digital technologies are being utilized intensively and extensively during the COVID-19 pandemic, older adults are largely excluded from both the real society and the virtual society because of the age-related digital divide.

#### 4.1.1. Physical Exclusion

On the one hand, older adults are at a disproportionate risk of severe infection and mortality. To avoid being infected, it is suggested that they reduce participation in social engagements and community gatherings. Physical contact, which is an important component of intimacy and reassurance in old age, has been discouraged [1]. This may exacerbate existing loneliness and significantly reduce quality of life. More importantly, without access to the Internet and/or without the capability to use digital devices, older adults cannot take public transportation, reserve medical care services, or even maintain their daily life during the pandemic. As shown in Case 8, some older adults cannot order food at a restaurant, as dishes need to be ordered by scanning a code and payment must be made with digital payment. Some cannot pay for utility bills or make online appointments, as digital payment instead of banknotes is the only option, as indicated in Cases 5 and 9. Rapid digitalization, together with the intensive and extensive application of information technology during the pandemic, has physically excluded older adults from real life in many aspects.

#### 4.1.2. Virtual Exclusion

On the other hand, older adults have inadequate media and information literacy and skills compared to other age cohorts [80,100]. They prefer to collect information and complete daily tasks via traditional ways, such as making an appointment on site in the hospital rather than booking it online, as indicated in Case 4 and Case 5. However, due to the pandemic, many daily tasks have to be conducted online and social media becomes the most convenient and prevalent way for obtaining access to instant information. However, due to media and information illiteracy, older adults use digital technologies less and hence receive less information during the pandemic [94,104,105,106]. More particularly, older adults may have difficulty in using digital technologies to conduct virtual interactions, including receiving the latest online news, video or voice chatting with friends and family members, seeking support from the Internet, and engaging in public events through social media. For instance, some medical care institutions provide remote medical care consultation services, aiming to give more options to patients who are unable to come to the institutions physically. However, due to the age-related digital divide, older adults are not the principal beneficiary of this type of service. The population which is supposed to receive more medical support during the pandemic is, in fact, not receiving enough help. The feeling of alienation and being out of touch would deteriorate the wellbeing of the old adults [1,113,114].

### 4.2. Causes of Age-Related Digital Divide during the COVID-19 Pandemic

Firstly, consistent with existing research, personal factors including education and income, attitudes, and motivations do play a role in shaping the age-related digital divide [6,7,88,115]. However, the COVID-19 pandemic has exacerbated the divide. Codes and other information technology have been utilized extensively during the pandemic, such as for making online appointments for medical care services, ordering and paying for food at a restaurant, or booking a taxi in advance for travel. However, some older adults do not know about the sudden changes, as shown in Cases 1, 2, and 4. They are confused, vulnerable and struggled to deal with information technology or devices. Nevertheless, some older adults are aware of the convenience of using digital technologies, but they are still reluctant to use them. An extra financial burden, caused by buying and using smartphones, is perceived as an obstacle for them to adopt digital technologies. Secondly, governmental policies significantly influence the age-related digital divide. Although using digital technologies, particularly digital codes, is an effective and efficient way to control the pandemic, making digital technologies the only option certainly does not consider the existing digital divide among different groups of people. As a disadvantaged population group, many older adults do not have smartphones, as shown in Case 1 and 4. They have no access to smartphones or the Internet, which makes obtaining digital codes and/or online services impossible. However, in the early days of the COVID-19 pandemic, many local governments made digital codes the only valid licenses for individuals to be engaged in any outdoor activities. This policy has certainly deprived older adults’ basic rights.

At the same time, inadequate social support makes it more difficult for older adults to address the digital divide issues under the COVID-19 pandemic. Most older adults do not have much experience in using digital technologies, even if they have access to smartphones and the Internet, as shown in Cases 2, 3, 5, 6, 8, and 9. They need technical and emotional support dealing with the age-related digital divide from not only their family, but also society. However, as the pandemic is unprecedented, the massive utilisation of information technology happened in a very short period. It is almost impossible for any institution to provide digital literacy training for older people. Most older adults could only seek support from family members, who are indeed the primary helpers for older adults during the pandemic. However, family support is not always adequate or timely, as shown in Cases 3, 5, and 9. Moreover, those older adults who live alone certainly suffer more than their counterparts living with their family members during the pandemic.

During the pandemic, there has been an intensive and extensive digital technology utilisation, with impacts on all aspects of work and life. Due to an age-related digital divide, older adults face more challenges and difficulties. In this paper, we analysed nine cases reported by the media concerning the age-related digital divide in three scenarios. We argue that personal attitudes and motivations, as well as education and income, governmental policies, and family and social support, are all major contributors to the severe impacts of the age-related digital divide on older adults during the pandemic. Because of the age-related digital divide, older adults are facing higher risks of being excluded from both the real society and the virtual society. The double exclusions will further jeopardize their health and reduce their life quality.

## 5. Policy Implications and Future Research

The age-related digital divide has been widening during the pandemic. As one of the groups most represented in the information-poor population, older adults suffer from more challenges and difficulties than other age cohorts [1,106]. Acknowledging such challenges facing by older adults, the government has paid much more attention and expended efforts to address this issue. In November 2020, the Chinese government issued a special policy aiming to support older adults addressing difficulties and challenges when using various digital technologies. There are plans to establish a long-term effective mechanism to bridge the age-related digital divide. In order to achieve this goal, the government needs to adopt more supportive measures to help older adults. Moreover, enterprises need to make new IT designs more suitable for older adults and update existing digital technologies to a senior-friendly design oriented to reduce the operational obstacles for older adults to use different types of digital devices. With regard to pandemic preventions and controls, considering the pandemic may last for a long period of time, digital technologies, especially digital codes, will remain a major measure to control the spread of the virus in China. However, digital codes should not and cannot be the only choice for such preventive and control measure. Paper certificates or other alternative certificates, such as ID cards and security cards, should be used as substitutes. However, the problem of the age-related digital divide is not just a technical issue but more a social issue. Families and communities need to provide more support for older adults both technically and emotionally to help older adults reduce technology anxiety and gain more digital literacy and digital skills in the information society.

This research is not without limitations. Firstly, the cases collected in the three most affected scenarios cannot cover all the challenges and difficulties older adults are facing during the pandemic. The cases examined in this research represent only a small portion of unpleasant and uncomfortable situations that older adults suffer in daily life during the pandemic. Secondly, the case studies were conducted based on limited information released by the media. The digital divide is highly related to age, but it also cuts across other areas including gender, job experience, social status, etc. However, with limited information regarding other areas for either privacy concern or out of censorship, we can draw no substantial conclusions regarding the role of intersectional issues. Further research examining more intersectional factors and exploring how to narrow the age-related digital divide through either interviews or questionnaire surveys should be conducted to achieve a better understanding of the age-related digital divide.

## Figures and Tables

**Table 1:** Categories and meanings of health codes.

Health Code Category by Colour	Meaning of the Code Colour
Red	In the past 14 days, the individual has been a close contact of an infected individual or has COVID-19 symptoms. Fourteen-day quarantine at a mandatory site or at home is compulsory. Daily reports about health status, such as body temperature, via the health code system is required. Individuals with a red code are forbidden to travel before the code turns green.
Yellow	In the past 14 days, the individual is likely to have been exposed to the virus. Seven-day quarantine at home is compulsory. The yellow health code’s requirements for daily health status report and travel restrictions are the same as those of the red health code.
Green	An individual with a green code is allowed to travel within and to low-risk or risk-free areas. No quarantine is needed.

Source: National and provincial policies on pandemic prevention and control in China.

**Table 2:** Summary of Governmental Policies Regarding Three Scenarios.

Scenarios	Strict Policies Implemented Nationwide during the Pandemic	Special Policies Issued by the State in November 2020 to Support Older Adults
*Transportation* (Health code, digital payment and online booking are required)	Only a valid health code or pass code (green) is accepted as travel permit.	Health code should not be the only legitimate travel permit. Alternative certificates such as ID cards and valid paper travel permits issued by the local authority should also be optional for the older adults.
	When taking public transportation, a valid health code (green) is compulsory.	Older people should be allowed to use ID cards, social security cards, and senior cards when they take city public transportation.
	Public areas such as tourist attractions, cultural centres, and museums need to be reserved online in advance. Train tickets needs to be booked online.	For public places whose their access needs to be reserved, manual service desks and telephone lines should be available. A certain number of onsite ticket quotas should be reserved for older people.
Medical Care (Health code, online booking and digital payment are required)	All the onsite appointment desks are cancelled except for emergencies and fever clinic. Appointments can be only made through internet or telephone.	Medical institutions should provide multiple choices for older people to make an appointment and keep a certain number of appointments available on site. Medical institutions should keep service desks for registration, payment, and printing the test reports. Volunteers, social workers, and other personnel should be available to help older people.
	Individuals without appointments and valid health codes or pass codes (green) are not allowed to enter medical care institutions.	Online medical care process should be simplified; voice instruction and in-person counselling for older people should be provided. Medical institutions should allow older people to use their ID cards, social security cards and other certificates when they take medical care services. Face recognition technology is encouraged to be adopted in medical institutions.
Consumption (Online ordering and digital payment are required)	In the lockdown situation, goods can only be purchased or ordered online, and digital payment is required.	No shops and individuals can refuse payment in cash. Cash and bank cards should be accepted in popular consumption places for older people, such as stores, markets, restaurants, parks, electronic and water bill payment units, as well as other administration bill payment units.

Source: National and provincial policies on pandemic prevention and control in China.

**Table 3:** Profiles of 9 cases from top 3 official media in China (February 2020–February 2021).

Scenarios	Situations	Challenges and Difficulties	Case Description	Quotes from the Interviewee
Transportation	1. Valid health code is required for taking public transportation and getting access to public areas. 2. Many public areas including parks, museums, libraries, galleries, gyms, need to be reserved and paid online in advance due to the pandemic control.	1. Unable to enter any public area without health code; 2. Unable to book ticket online; 3. Unable to take any public transportation.	Case 1: An older man was not allowed to enter the subway station without a health e-code, and he had a quarrel with station staff. (People.cn 18. Aug 2020) [19]	“Why am I not able to enter the station? What is the health e-code? I don’t know about it. No one gives it to me.”
			Case 2: Ms. Li, a 64-year-old female, failed to enter the hospital and afterwards failed to get a taxi because she could not produce her health code. (Xinhuanet, 4 Jan 2021) [20]	“I used my finger to swipe up and down on screen, but the health code just didn’t show up.” “No passenger in the car, why don’t they (taxis) stop?”
			Case 3: Mr. Li in his 60s hailed a taxi in the street. However, all the taxis running in the street were already booked by others via apps. After waiting in vain for one hour, he had to call his family for help. (Xinhuanet, 3 Sep 2020) [21]	“Many things have to be handled online, but I don’t know how to use the software in my mobile phone. So I have to find someone for help.”
Medical Care	1. Appointment needs to be made online or through self-service machines onsite, and only digital payment is accepted for online appointment. 2. Only individuals with valid health e-codes or pass codes are allowed to enter medical care institutions.	1. Unable to make online appointment; 2. Unable to enter any medical institution; 3. Unable to search for health care information online.	Case 4: Mr. and Ms. Wei did not know that they need to make an appointment in advance until they arrived at the hospital in the early morning. They had to make an appointment through a self-service machine or a smartphone. (GMW, 21 Sep 2020) [22]	“My wife has a gastrointestinal problem. We came here by bus very early in the morning but couldn’t even manage to make an appointment.”
			Case 5: Ms. Li, 78 years old, had to make an appointment online by using the self-service machine in the hospital. However, she did not know how to use the machine and she did not have any digital payment code. The appointment was finally made with the help of a young girl in the queue. (Xinhuanet, 6 Oct 2020) [23]	“An Online appointment is required everywhere. I am not able to do it. Except for answering phone calls, I could not use any other applications on my phone.”“They (her two sons) both have their sons, and they are very busy taking care of them (her grandsons). They even have to work during holidays. How could I count on them (come to the hospital) to make an appointment for me?”
			Case 6: Mr. Zhang, 86 years old, complained that although there were volunteers in the hospital, it was not easy for someone at his age to use the self-service machine or payment code. (Xinhuanet, 12 Nov 2020) [24]	“Sometimes there are lots of people waiting to use self-service machines to make appointments. I have poor eyesight, couldn’t see clearly, and worried about being hurried by others, (also worried about) pressing wrong buttons. ”
Consumption	1. Due to social distancing, many supermarkets and restaurants only serve consumers online. 2. Payment codes are utilized in various places. Some shops and restaurants do not accept notes or coins. Digital payment is the only choice.	1. Unable to purchase goods online; 2. Unable to pay for goods or bills either online or offline.	Case 7: Ms. Yang in her 70s went to see a doctor. She spent more than half an hour to complete the registration process through self-service machine.(People.cn, 6 Dec 2020) [25]	“Nowadays, you have to scan codes to complete the payment for food. Cash is becoming less acceptable. It is very inconvenient if you do not use digital payment. ”
			Case 8: Mr. and Ms. Chen both in their 70 s, had no experience in using APPs on their phones. They spent more than half an hour figuring out using QR code to order food in a restaurant without getting any help. (People.cn, 1 Feb 2021) [26]	“Now when we go to a restaurant and find that the order has to be done by scanning the QR code, we feel uncomfortable.”
			Case 9: Ms. Wu came to pay her electricity bill early in the morning and asked a young man to show her how to use her smartphone to complete the payment. (Xinhuanet, 10 Sep 2020) [27]	“I am getting older and learning it (using digital payment) very slowly. I may forget next time. I am lucky to find someone to teach me, but my brother who is in his 70s, lives alone in the remote mountain. It is difficult for him to receive supports. ”

## Data Availability

For the links to the collected cases, please refer to the references.
